# TMPRSS2 Expression in Lung Tissue of Prostatic Adenocarcinoma Patients: Androgen Deprivation Therapy and Relevance to SARS-CoV-2 Infection

**DOI:** 10.3390/cimb47100823

**Published:** 2025-10-08

**Authors:** Marcela Riveros Angel, David Loeffler, Ahmad Charifa, Ryan B. Sinit, Taylor Amery, Beyza Cengiz, Tomasz M. Beer, George V. Thomas

**Affiliations:** 1 Department of Pathology & Laboratory Medicine, Oregon Health & Science University, Portland, OR 97201, USA; 2 Department of Oncology, Oregon Health & Science University, Portland, OR 97201, USA; 3 Knight Cancer Institute, Oregon Health & Science University, Portland, OR 97201, USA

**Keywords:** SARS-CoV-2, androgen receptor (AR), TMPRSS2, prostate, androgen deprivation therapy (ADT)

## Abstract

Severe acute respiratory syndrome coronavirus 2 (SARS-CoV-2) cellular entry is facilitated by transmembrane protease serine 2 (TMPRSS2), which is regulated by the androgen receptor (AR). Androgen deprivation therapy (ADT), widely used in prostate cancer treatment, may potentially modulate TMPRSS2 expression, affecting SARS-CoV-2 infection susceptibility and severity. We evaluated the impact of ADT on pulmonary TMPRSS2 expression in prostate cancer patients and analyzed differences in expression patterns associated with specific ADT regimens. We examined TMPRSS2 immunohistochemical expression in lung tissue from 20 consecutive autopsy cases of men with prostate cancer (6 receiving ADT at time of death), compared with non-ADT prostate cancer patients and age-matched women controls. Histoscores were calculated by assessing the percentage and intensity of pneumocyte TMPRSS2 expression. Prostate cancer patients receiving ADT showed significantly reduced pulmonary TMPRSS2 expression compared to non-ADT patients (mean histoscores: 152.7 vs. 225.0, *p* = 0.037) and age-matched women controls (mean histoscores: 152.7 vs. 238.0, *p* = 0.024). Direct AR antagonists (apalutamide, bicalutamide) produced greater TMPRSS2 suppression than Gonadotropin-Releasing Hormone modulators or androgen biosynthesis inhibitors. No significant correlation was observed between the TMPRSS2 expression and Gleason score, PSA levels, or underlying lung pathology. Our findings demonstrate that ADT significantly reduces pulmonary TMPRSS2 expression, with direct AR antagonists showing the strongest effect. This suggests a potential mechanistic explanation for differential COVID-19 susceptibility and provides a rationale for investigating AR-targeted therapies as potential protective interventions against SARS-CoV-2 infection severity.

## 1. Introduction

The coronavirus disease 2019 (COVID-19) pandemic, caused by severe acute respiratory syndrome coronavirus 2 (SARS-CoV-2), continues to pose significant global health challenges despite advances in prevention and treatment. According to the World Health Organization, COVID-19 has resulted in over 7 million deaths worldwide as of early 2025, highlighting the ongoing importance of understanding the viral pathogenesis and identifying effective interventions.

SARS-CoV-2 cellular entry involves a coordinated multi-step process. The viral spike (S) glycoprotein undergoes initial priming by furin, followed by binding to the angiotensin-converting enzyme 2 (ACE2) receptor on host cell surfaces [1,2]. Upon ACE2 binding, two distinct entry pathways are utilized: the primary pathway involves the cleavage of the S2′ subunit by transmembrane protease serine 2 (TMPRSS2), exposing the viral fusion peptide and facilitating membrane fusion; alternatively, in cells with low TMPRSS2 expression, the virus can be internalized via endocytosis, with cathepsin L mediating the S protein cleavage within endosomes [1,3,4,5,6]. This mechanistic understanding has prompted investigations into targeting these entry factors as potential therapeutic approaches.

TMPRSS2, a key facilitator of SARS-CoV-2 entry, is transcriptionally regulated by the androgen receptor (AR) and is expressed in multiple tissues, including the prostate, lung, colon, and kidney [7]. This androgen dependence has attracted particular attention given the significant sex disparity in COVID-19 mortality, with men experiencing approximately 1.5-fold higher death rates across all age groups. In prostate cancer, TMPRSS2 gene alterations serve as important biomarkers, with TMPRSS2-ETS gene fusions (particularly TMPRSS2-ERG) occurring in approximately 50% of cases and associated with aggressive disease features [8,9,10,11,12,13,14,15]. While the complete implications of these fusions remain incompletely understood, they contribute to tumor invasion, angiogenesis, and androgen independence.

Androgen receptor signaling drives prostate cancer progression, making androgen deprivation therapy (ADT) the foundation of treatment for metastatic disease. Contemporary ADT approaches include Gonadotropin-Releasing Hormone analogs (leuprolide, degarelix), direct AR antagonists (bicalutamide, enzalutamide, apalutamide, darolutamide), and androgen biosynthesis inhibitors (abiraterone and ortreonel) [16,17,18]. The connection between AR-regulated TMPRSS2 expression and SARS-CoV-2 entry has prompted investigations into whether ADT might confer protection against COVID-19 severity in prostate cancer patients, though results have been inconsistent [19].

Recent work by Schuler et al. demonstrated developmental increases in pulmonary TMPRSS2 expression, with prominent expression in secretory, ciliated, and alveolar type 1 epithelial cells. Their analysis of COVID-19 autopsy specimens revealed higher viral infection rates in TMPRSS2-expressing cells, further supporting its role in the viral pathogenesis [4]. Similarly, Samuel et al. identified associations between elevated free androgen levels and COVID-19 complications in autopsy studies [5].

The present study was designed to determine whether ADT modulates TMPRSS2 expression in the lung tissue of prostate cancer patients and to assess whether specific ADT regimens differ in their effects on pulmonary TMPRSS2 levels. We hypothesized that ADT would reduce TMPRSS2 expression in lung tissue, potentially providing a mechanistic explanation for differential COVID-19 susceptibility. Through the immunohistochemical analysis of autopsy specimens, we demonstrate a significant reduction in pulmonary TMPRSS2 expression in ADT-treated patients compared to untreated prostate cancer patients and controls, with direct AR antagonists showing particularly potent suppressive effects. These findings offer novel insights into the regulation of this key viral entry factor and suggest potential therapeutic strategies for mitigating SARS-CoV-2 infection severity.

## 2. Materials and Methods

### 2.1. Patient Selection and Data Collection

Following review by institutional review board, the OHSU IRB determined that the proposed activity (Study ID# 00021427) involved the use of autopsy tissues and therefore is not research involving human subjects. Consequently, IRB review and approval is not required. We conducted a retrospective analysis of consecutive autopsy cases from our institution (Oregon Health & Science University) between 2010 and 2019. Using the Epic SlicerDicer tool, we identified 20 male patients with documented prostatic adenocarcinoma. For each case, we retrieved comprehensive clinical information including demographics, medical history, treatment details, and laboratory values from electronic health records.

We selected lung tissue slides from the pathology archives for assessment. Exclusion criteria included completely autolyzed tissue or pneumonia-involved tissue lacking normal lung parenchyma as control. We established a control group of 10 age-matched women patients who underwent autopsy during the same period, enabling comparative analyses while accounting for age-related histological changes.

Clinical parameters documented for all cases included age at death, race, smoking history, comorbidities, cause of death, and medications. For prostate cancer patients, we additionally recorded Gleason score, treatment history, PSA levels at time of death, LDH, serum testosterone, and ADT regimen, as summarized in Table 1.

### 2.2. Histopathologic Evaluation

Two independent board-certified surgical pathologists, blinded to clinical data, performed thorough assessment of pathological alterations in all specimens. Pathological findings were documented and cross-referenced with diagnoses from clinical records and autopsy reports. This comprehensive approach allowed correlation of microscopic features with clinical information, including metastases, primary lung pathology, and other significant findings (Table 2).

### 2.3. Immunohistochemical Analysis

Immunohistochemical staining was performed on 4 μm formalin-fixed, paraffin-embedded tissue sections using anti-TMPRSS2 antibody (clone EPR3861, dilution 1:6000; ab92323, Abcam, Waltham, MA, USA) on a Benchmark-Ultra automated system (Ventana-Roche, Tucson, AZ, USA). The specificity of the antibody was validated using appropriate positive and negative controls.

### 2.4. TMPRSS2 Expression Assessment

TMPRSS2-stained slides were digitally scanned at high resolution and independently analyzed by two pathologists blinded to clinical data. For each specimen, the percentage of pneumocyte staining was assessed, and nuclear staining intensity was categorized on a scale of 0 to 3 (0 = none, 1 = weak, 2 = moderate, 3 = strong). A histoscore was calculated for each sample using the formula (1 × [% cells with 1+ staining] + 2 × [% cells with 2+ staining] + 3 × [% cells with 3+ staining]), yielding scores ranging from 0 to 300, with higher values indicating greater TMPRSS2 expression.

### 2.5. Statistical Methods

Statistical analyses were performed using GraphPad Prism software (Version 10). TMPRSS2 histoscores between groups were compared using the Kolmogorov–Smirnov test, which evaluates differences in cumulative distributions without making assumptions about underlying data distribution. This non-parametric approach was selected to accommodate the variable distributional characteristics inherent to immunohistochemical scoring data and to maintain statistical validity with the modest sample sizes typical of consecutive autopsy studies.

All data points were retained in the analysis without outlier removal, as extreme histoscore values were considered to represent genuine biological variation rather than technical artifacts. The Kolmogorov–Smirnov test was applied to compare the complete distributional profiles between treatment groups (ADT vs. no ADT), disease status (prostate cancer vs. normal controls), and combined categories as appropriate.

No post hoc corrections for multiple comparisons were applied, as comparisons were hypothesis-driven and focused specifically on the biological question of ADT effects on pulmonary TMPRSS2 expression. Statistical significance was set at *p* < 0.05. Results are presented as individual data points with median values, allowing visualization of the complete distributional patterns that informed the Kolmogorov–Smirnov comparisons

## 3. Results

### 3.1. Patient Characteristics

Our cohort comprised 20 deceased men with prostate adenocarcinoma, all identified as white, with ages ranging from 45 to 86 years (median 68.5). Smoking history varied across the cohort, including never smokers, former smokers, and current smokers. All patients presented with comorbidities, most commonly hypertension, diabetes mellitus, and cardiovascular disease (Table 1).

Of the 20 prostate cancer patients, 6 were receiving ADT at time of death (leuprolide, bicalutamide, degarelix, orteronel, apalutamide, or abiraterone), ranging in age from 60 to 83 years (median 78). All six had disseminated metastatic castration-resistant prostate cancer (CRPC), with bone being the most common metastatic site (n = 6), followed by lymph nodes (n = 4) and other organs (n = 2). Common causes of death included hemorrhagic complications (n = 2), cardiovascular events (n = 1), bowel obstruction (n = 1), and direct complications of metastatic disease (n = 2).

Among the 14 remaining patients, 10 had no documented history of ADT, while the ADT status could not be confirmed in 4 patients. The documented non-ADT and uncertain ADT group ranged in age from 45 to 86 years (median 68.5), with a median Gleason Score of six (Grade Group 1). Primary causes of death included cardiovascular/cerebrovascular events, traumatic hemorrhage, and respiratory complications.

The women control group (n = 10) ranged in age from 46 to 86 years (median 66), with cardiovascular disease being the most common cause of death.

### 3.2. Pulmonary Histopathologic Findings

**Figure 1:**
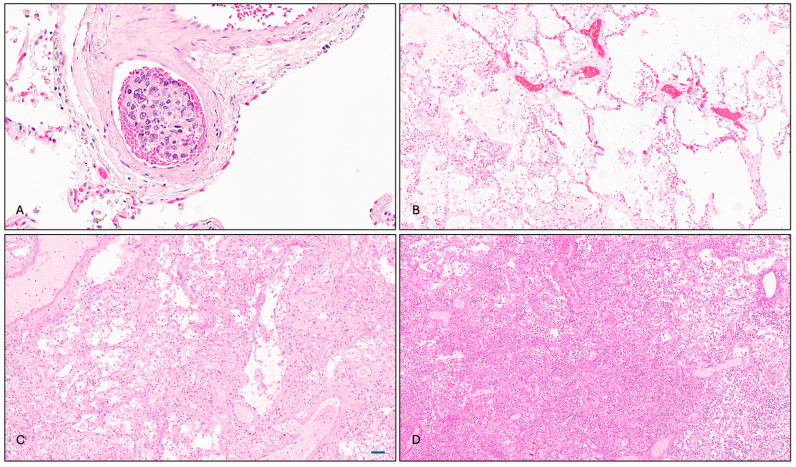
Representative pulmonary histopathology (hematoxylin–eosin stain). (**A**) Pulmonary infiltrates of metastatic prostatic adenocarcinoma with tumor emboli in vascular spaces. (**B**) Emphysematous changes with enlarged distal airspaces and alveolar septal destruction. (**C**) Acute organizing pneumonia with alveolar macrophage accumulation and fibrin deposition. (**D**) Bronchopneumonia with neutrophilic infiltration and proteinaceous exudates (original magnification: (**A**) ×200, (**B**–**D**) ×100). Scale bar (72 μm).

The pathological examination revealed that two ADT-treated patients who died from metastatic CRPC exhibited pulmonary infiltrates of prostatic adenocarcinoma, manifesting as intravascular tumor thrombi (Figure 1A). While these findings likely contributed to clinical deterioration, they were not deemed the principal cause of death. Additional pulmonary findings in the ADT-treated group included emphysematous changes, vascular congestion, multifocal pneumonia, arterial thickening, and fibrin thrombus formation (Figure 1B).

Patients in the non-ADT and uncertain ADT groups presented with diverse pulmonary histopathologies, most commonly emphysematous changes and congestion (5 out of 14), followed by inflammatory and infectious features (5 out of 14), including alveolar macrophage accumulation, acute pneumonia, and bronchial bacterial/fungal pneumonia. Bronchopneumonia was the adjudicated cause of death in one case (Figure 1C). In the control group, 8 out of 10 subjects showed underlying pulmonary disease at time of death, with bronchopneumonia being the most frequent (Figure 1D).

### 3.3. TMPRSS2 Expression Analysis

**Figure 2:**
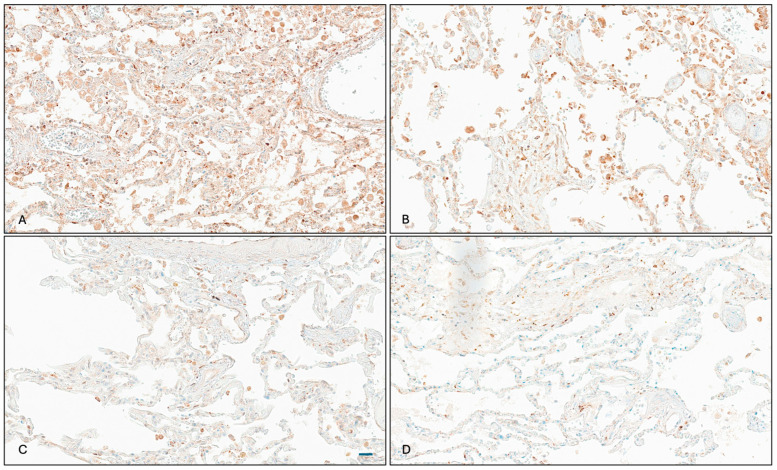
TMPRSS2 immunohistochemistry in lung tissue. (**A**,**B**), lung tissue from prostate cancer patients not treated with ADT showing strong TMPRSS2 expression (histoscores 221.1 and 277.5, respectively, Scale bar (72 μm)). (**C**,**D**), lung tissue from ADT-treated prostate cancer patients showing markedly reduced TMPRSS2 expression (histoscores 90.0 and 107.5, respectively) (original magnification ×200). Abbreviations: TMPRSS2, transmembrane protease serine 2.

The immunohistochemical analysis revealed TMPRSS2 expression primarily in alveolar pneumocytes, with a varying intensity across patient groups. The quantitative assessment showed significantly reduced TMPRSS2 protein expression in prostate cancer patients receiving ADT compared to those not receiving ADT (mean histoscores: 152.7 vs. 225.0, *p* = 0.037) (Figure 2A–D). When including patients with an uncertain ADT status, the trend persisted but narrowly missed statistical significance (*p* = 0.058).

**Figure 3:**
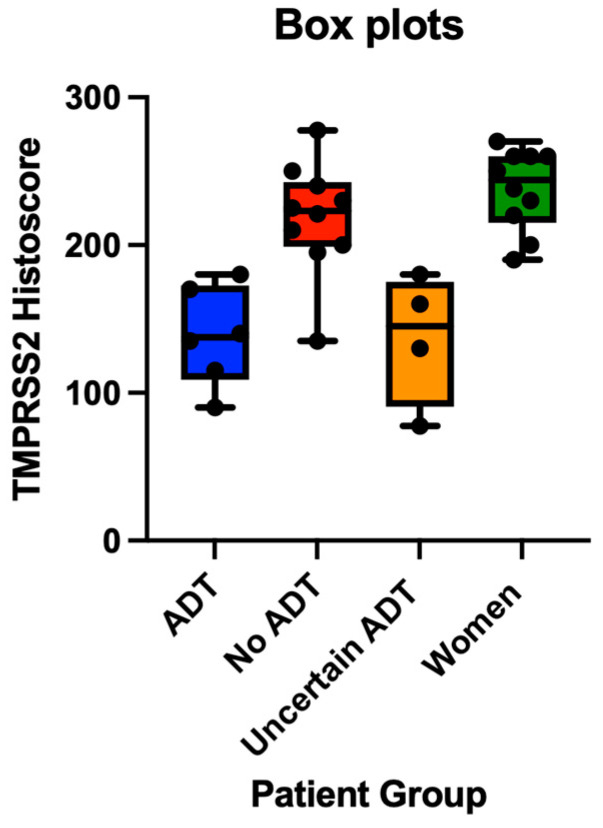
TMPRSS2 expression by patient group. Box plot showing distribution of TMPRSS2 histoscores across ADT-treated prostate cancer patients (n = 6), non-ADT prostate cancer patients (n = 10), patients with uncertain ADT status (n = 4), and women controls (n = 10). Boxes represent interquartile range, horizontal lines indicate median values, squares show means, and whiskers extend to minimum and maximum values within 1.5 × IQR. Individual data points are plotted as circles. TMPRSS2 expression was significantly reduced in ADT-treated patients compared to non-ADT patients (*p* = 0.037) and women (*p* = 0.024). Abbreviations: ADT, androgen deprivation therapy; AR, androgen receptor; and TMPRSS2, transmembrane protease serine 2.

Notably, TMPRSS2 expression was also significantly lower in ADT-treated patients compared to women controls (mean histoscores: 152.7 vs. 238.0, *p* = 0.024), while the expression was comparable between non-ADT men and women controls (*p* = 0.164) (Figure 3). Patients with an uncertain ADT status also displayed relatively low TMPRSS2 expression (mean: 136.9), potentially indicating a mixed population including some previously treated individuals.

**Figure 4:**
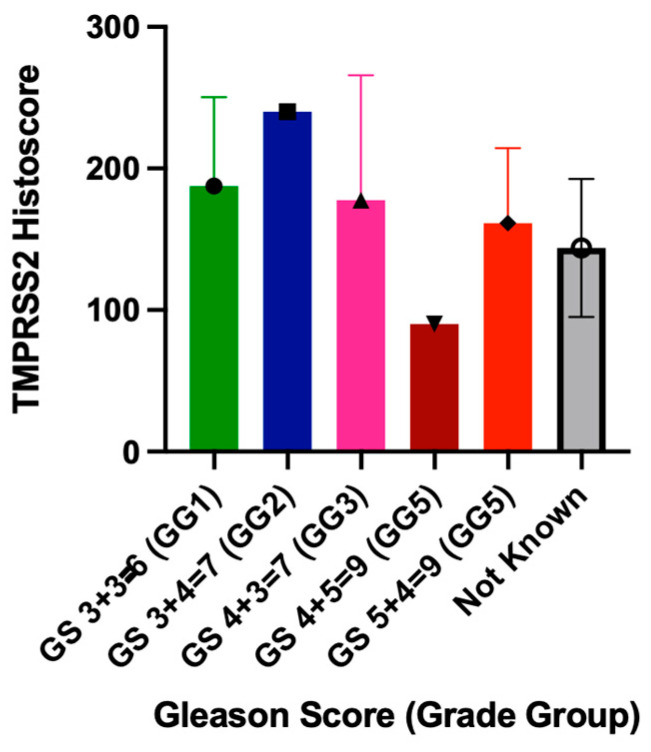
TMPRSS2 expression in lung tissue stratified by prostate tumor Gleason score. No significant correlation was observed, with Gleason 6 cases (n = 8) showing wide variability (mean: 187.5; range: 107.5–277.5). Higher-grade tumors did not consistently show elevated expression, indicating that Gleason score does not predict pulmonary TMPRSS2 levels.

We observed no significant correlation between TMPRSS2 expression and prostate tumor Gleason scores (Figure 4). Gleason 6 cases (n = 8) showed a wide variation in TMPRSS2 histoscores (range: 107.5–277.5, mean: 187.5), while higher-grade tumors did not consistently exhibit higher TMPRSS2 expression. The absence of a linear relationship between the Gleason grade and TMPRSS2 histoscores suggests that tumor differentiation alone does not predict pulmonary TMPRSS2 expression.

Similarly, no clear correlation emerged between PSA levels and TMPRSS2 expression in ADT-treated patients, though the sample size for this analysis was limited (n = 5). Patients with remarkably high PSA levels (>200 ng/mL) generally exhibited lower TMPRSS2 expression (histoscores ~90–160), while one patient with lower PSA (13.77 ng/mL) demonstrated higher expression (histoscore 240). The presence of underlying lung disease was associated with a modest, non-significant reduction in TMPRSS2 expression (mean histoscores: 180.1 vs. 194.1, *p* = 0.62).

### 3.4. Effect of ADT Agent Type on TMPRSS2 Expression

**Figure 5:**
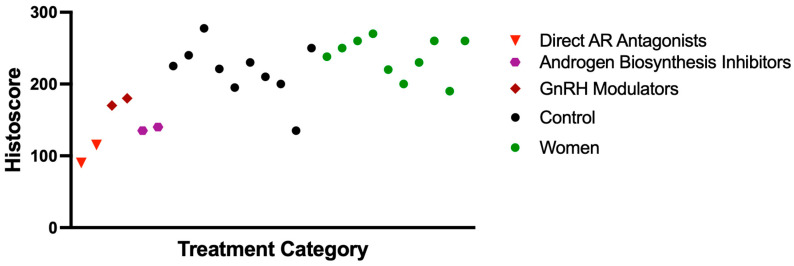
TMPRSS2 expression by specific ADT regimen. Individual patient histoscores grouped by ADT mechanism: Direct AR antagonists (bicalutamide + leuprolide, apalutamide + leuprolide), GnRH modulators (leuprolide monotherapy, degarelix), androgen biosynthesis inhibitors (abiraterone + leuprolide, orteronel + degarelix), and control groups (no ADT, women). Direct AR antagonists produced the most pronounced reduction in TMPRSS2 expression. Abbreviations: ADT, androgen deprivation therapy; AR, androgen receptor; TMPRSS2, transmembrane protease serine 2; and GnRH, gonadotropin-releasing hormone.

**Figure 6:**
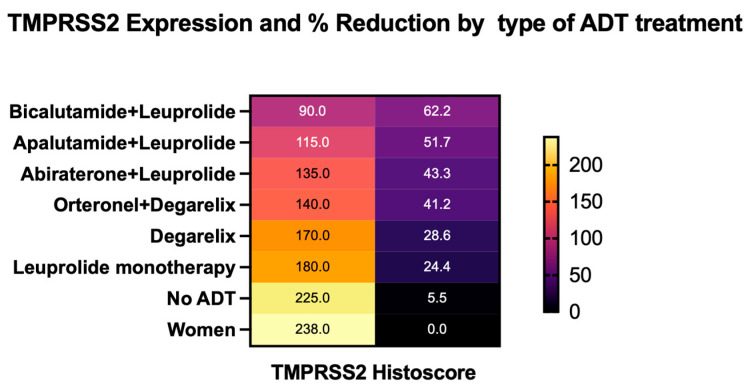
Relationship between TMPRSS2 expression and percent reduction by ADT regimen. Heat map simultaneously visualizing both absolute histoscores (left) and percent reduction from control levels (right) across ADT regimens. Darker intensity represents lower values. Direct AR antagonists (bicalutamide + leuprolide, apalutamide + leuprolide) show the lowest histoscores and highest percent reductions. Abbreviations: ADT, androgen deprivation therapy; AR, androgen receptor; TMPRSS2, transmembrane protease serine 2; and GnRH, gonadotropin-releasing hormone.

The analysis of TMPRSS2 expression across different ADT regimens demonstrated variable effects depending on the mechanism of action. The most substantial reductions were observed with direct androgen receptor (AR) antagonists (specifically bicalutamide and apalutamide), with mean histoscores of 90 and 115. These effects were even more pronounced when direct AR antagonists were combined with GnRH modulators, suggesting a potential synergistic impact. In contrast, GnRH modulators alone (leuprolide: 180; degarelix: 170) and androgen biosynthesis inhibitors (abiraterone: 135; orteronel: 140) were associated with more modest decreases in TMPRSS2 expression (Figure 5). These findings indicate that direct AR blockade, particularly in combination with upstream hormonal suppression, may more effectively reduce TMPRSS2 expression by targeting multiple points in the androgen signaling pathway (Figure 6).

## 4. Discussion

The COVID-19 pandemic has stimulated intensive research into SARS-CoV-2 cellular entry mechanisms and potential therapeutic targets [1,3,20,21]. TMPRSS2, a serine protease critical for S protein processing, has emerged as a key facilitator of viral entry into host cells. Upon ACE2 binding, SARS-CoV-2 undergoes conformational changes enabling the TMPRSS2-mediated cleavage of the S2′ subunit, exposing the fusion peptide and initiating membrane fusion [21]. The androgen-dependent regulation of TMPRSS2 and the male predominance in COVID-19 mortality have prompted investigations into androgen-targeted interventions as potential therapeutic strategies.

Our study provides novel evidence that androgen deprivation therapy in prostate cancer patients significantly reduces TMPRSS2 expression in lung tissue. This reduction was most pronounced with direct AR antagonists, suggesting differential efficacy based on therapeutic mechanisms. These findings have several important implications for understanding the COVID-19 pathogenesis and developing targeted interventions.

### 4.1. Impact of ADT on TMPRSS2 Expression and COVID-19 Outcomes

Recent studies have shown that TMPRSS2 expression in lung epithelial cells is modulated by androgen receptor (AR) signaling, providing a plausible mechanism for the observed suppression in patients receiving ADT. For example, Baratchian et al. demonstrated that dihydrotestosterone (DHT) stimulation upregulates TMPRSS2 in human bronchial epithelial cells (HBECs), while AR antagonism reduces its expression [22]. Similarly, Samuel et al. and Qiao et al. used human airway epithelial cells and lung organoids to confirm that AR activity directly influences TMPRSS2 levels and that AR inhibition attenuates viral entry in vitro [5,6]. These studies support the concept that TMPRSS2 regulation in lung tissue is at least partially autonomous from systemic androgen levels and can be modulated by direct AR-targeted therapies.

Given our findings that direct AR antagonists result in more pronounced TMPRSS2 suppression in lung tissue compared to GnRH modulators or androgen biosynthesis inhibitors, it would be valuable to compare these regimens in lung epithelial or organoid models. Such approaches would allow for the mechanistic dissection of how different ADT classes influence TMPRSS2 transcription through AR signaling, chromatin remodeling, or co-regulatory interactions, independent of systemic endocrine feedback. Future studies using these platforms could provide a more granular understanding of AR-targeted strategies for modulating viral entry proteins in the lung.

Earlier studies investigating the relationship between ADT and COVID-19 outcomes have yielded mixed results. Montopoli et al. reported significantly lower SARS-CoV-2 infection rates in ADT-treated prostate cancer patients compared to untreated patients in a large Italian cohort, suggesting potential protective effects [23]. Conversely, Shah et al. found no significant differences in hospitalization rates, oxygen requirements, or mortality between ADT-treated and untreated COVID-19 patients [24], while Duarte et al. observed no association between ADT use and reduced mortality in hospitalized Brazilian patients [25].

Our findings provide a potential mechanistic explanation for these discrepancies by showing that the effect on TMPRSS2 expression varies substantially based on specific ADT regimens. The most pronounced suppression was observed in patients treated with direct AR antagonists (apalutamide, bicalutamide) in combination with GnRH modulators; no cases of AR antagonist monotherapy were included in this study. In contrast, GnRH modulators and androgen biosynthesis inhibitors alone showed more modest effects. This differential impact suggests that the potential protective benefit of ADT may depend on the therapeutic approach, possibly explaining the heterogeneous clinical outcomes observed across studies with varied treatment protocols.

Furthermore, our observation that combination therapy (AR antagonist plus GnRH modulator) produced particularly marked TMPRSS2 suppression suggests that multi-level androgen signaling inhibition may offer enhanced protective effects. This finding aligns with recent preclinical work by Deng et al., who demonstrated that androgen receptor inhibition attenuates spike-mediated viral entry in lung and prostate cells [26], and Leach et al., who showed that enzalutamide decreases TMPRSS2 expression and inhibits viral entry in human lung cells and mouse models [27].

### 4.2. Differential AR Pathway Inhibition and Its Impact on Pulmonary TMPRSS2 Expression

Combination therapy with direct AR antagonists (apalutamide, bicalutamide) seems to have a stronger effect on TMPRSS2 expression than GnRH modulators or androgen biosynthesis inhibitors. This is probably because the two types of drugs work in different ways. Direct AR antagonists attach to the androgen receptor, stopping it from activating and then transcribing AR target genes like TMPRSS2. GnRH modulators, on the other hand, work upstream to stop the body from producing testosterone, and androgen biosynthesis inhibitors stop the body from producing steroids through enzymes. However, both may not work as well in tissues that can produce or keep androgen activity on their own. Peripheral tissues, including the lung, include enzymes like 5α-reductase and 17β-hydroxysteroid dehydrogenase that let them create and break down androgens from circulating precursors. This means that local androgen signaling can continue even after systemic inhibition. It is known that lung epithelial cells, notably type II pneumocytes and the bronchial epithelium, have androgen receptors and respond to androgenic stimuli [28]. So, even if androgen levels in the body are lower, receptor activity may still be present until it is specifically blocked.

Baratchian et al. showed that blocking the AR in airway epithelial cell models is better at stopping TMPRSS2 expression than just stopping androgens [22]. This suggests that blocking local receptors has a stronger effect on viral entry targets in the lung. Additionally, evaluations of intracrine metabolism show that peripheral tissues, such as the lung, can produce active androgens from precursors. This supports local AR signaling and makes systemic treatments less effective on their own [29].

Collectively, these data substantiate our conclusions: direct AR antagonists, especially when combined with upstream suppression, more efficiently inhibit local AR-mediated transcription, including TMPRSS2, in lung tissue.

### 4.3. Therapeutic Implications and Future Directions

The significant reduction in pulmonary TMPRSS2 expression with AR-targeted therapy suggests several potential therapeutic applications. First, for prostate cancer patients at high risk of COVID-19 exposure or complications, regimens incorporating direct AR antagonists might offer dual benefits of oncologic control and potential viral protection. Second, our findings provide a rationale for investigating AR antagonists as an adjunctive therapy in high-risk non-cancer patients with COVID-19, particularly males with elevated androgen levels.

The differential efficacy of various ADT approaches also has important implications for clinical trial design. Future studies should stratify patients by specific ADT regimens rather than treating ADT as a homogeneous intervention. Additionally, the apparent synergistic effect of combined AR antagonism and Gonadotropin-Releasing Hormone (GnRH) modulation suggests that multi-target approaches may be more effective than single-agent interventions.

Our observation that direct AR blockade more effectively reduces TMPRSS2 expression than other approaches also provides insights into the molecular regulation of this key viral entry factor. This suggests that local, tissue-level androgen signaling may be more important than systemic androgen levels in regulating pulmonary TMPRSS2 expression, a finding that could inform the development of tissue-selective AR modulators with optimized pulmonary activity.

### 4.4. Study Limitations and Strengths

Several limitations should be acknowledged. First, our sample size was relatively small, particularly for the subgroup analyses by specific ADT regimens. Second, the retrospective nature precludes the establishment of causal relationships between ADT and TMPRSS2 expression. Third, as an autopsy study, our findings may not fully reflect TMPRSS2 expression patterns in living patients with a less advanced disease.

TMPRSS2 plays a critical role in SARS-CoV-2 entry by priming the spike protein, thus facilitating viral fusion with host cell membranes. Our findings of reduced pulmonary TMPRSS2 expression in ADT-treated patients may suggest a biological mechanism by which ADT could reduce initial viral infectivity. However, it is important to note that the COVID-19 pathogenesis is multifactorial, involving viral, host immune, and endothelial factors. Reduced TMPRSS2 may mitigate early infection but is unlikely to fully explain outcomes, especially in later inflammatory phases.

Despite these limitations, our study has important strengths. The use of matched controls and comprehensive clinicopathologic characterization allowed robust comparative analyses. The inclusion of patients on various ADT regimens permitted the assessment of differential effects based on therapeutic mechanisms. Finally, the direct examination of lung tissue through validated immunohistochemical methods provides more definitive evidence of TMPRSS2 expression patterns than peripheral blood markers or in vitro models.

## 5. Conclusions

This study demonstrates that androgen deprivation therapy (ADT) significantly reduces TMPRSS2 expression in the lung tissue of prostate cancer patients, with direct AR antagonists exerting the most pronounced effect. These findings offer a mechanistic rationale for the potential protective role of ADT against severe COVID-19 and highlight targeted AR inhibition as a possible strategy to mitigate SARS-CoV-2 infection. While the results are compelling, the limited number of ADT-treated cases (n = 6) constrains the statistical power and limits the ability to fully account for covariates such as comorbidities, smoking history, Gleason scores, and the tumor burden. Our study was limited to a histopathologic evaluation and did not include COVID-19 incidence or outcome data. Therefore, no direct correlation between pulmonary TMPRSS2 levels and the SARS-CoV-2 infection risk or clinical severity can be made. Expanding the cohort with a comprehensive clinical annotation will be essential to refine these associations and clarify the independent effect of ADT on pulmonary TMPRSS2 expression. Prospective, large-scale studies are warranted to validate our findings and determine whether TMPRSS2 suppression translates into measurable improvements in COVID-19 outcomes.

## Figures and Tables

**Table 1:** Patient demographics and clinical characteristics.

Characteristic	ADT Group (n = 6)	Non-ADT Group (n = 10)	Uncertain ADT Status (n = 4)	Women (n = 10)
**Age (years)**				
Median (range)	78 (60–83)	68.5 (45–86)	64 (61–74)	66 (46–86)
**Race**				
White	6 (100%)	10 (100%)	4 (100%)	10 (100%)
**Smoking History**				
Never smoker	3 (50%)	4 (40%)	1 (25%)	3 (30%)
Former smoker	3 (50%)	5 (50%)	2 (50%)	5 (50%)
Current smoker	0 (0%)	1 (10%)	1 (25%)	2 (20%)
**Gleason Score**				
6 (3 + 3)	1 (16.7%)	7 (70%)	0 (0%)	N/A
7 (3 + 4)	1 (16.7%)	0 (0%)	0 (0%)	N/A
7 (4 + 3)	1 (16.7%)	1 (10%)	0 (0%)	N/A
9 (4 + 5)	1 (16.7%)	0 (0%)	0 (0%)	N/A
9 (5 + 4)	1 (16.7%)	1 (10%)	0 (0%)	N/A
Unknown	1 (16.7%)	1 (10%)	4 (100%)	N/A
**PSA at Death (ng/mL)**				
Median (range)	149.6 (13.8–2308.5)	N/A	N/A	N/A
**ADT Regimen**				
Leuprolide + apalutamide	1 (16.7%)	N/A	N/A	N/A
Leuprolide + bicalutamide	2 (33.3%)	N/A	N/A	N/A
Degarelix + orteronel	1 (16.7%)	N/A	N/A	N/A
Leuprolide + abiraterone	1 (16.7%)	N/A	N/A	N/A
Leuprolide monotherapy	1 (16.7%)	N/A	N/A	N/A
**Metastatic Status**				
Bone	6 (100%)	0 (0%)	0 (0%)	N/A
Lymph node	4 (66.7%)	0 (0%)	0 (0%)	N/A
Other organs	2 (33.3%)	0 (0%)	0 (0%)	N/A
**Primary Cause of Death**				
Metastatic disease	2 (33.3%)	0 (0%)	0 (0%)	0 (0%)
Hemorrhagic complications	2 (33.3%)	2 (20%)	0 (0%)	1 (10%)
Cardiovascular event	1 (16.7%)	5 (50%)	2 (50%)	5 (50%)
Respiratory failure/pneumonia	0 (0%)	1 (10%)	0 (0%)	3 (30%)
Other	1 (16.7%)	2 (20%)	2 (50%)	1 (10%)

**Table 2:** Summary of pulmonary histopathologic features.

Histopathologic Finding	ADT Group (n = 6)	Non-ADT Group (n = 14)	Women Controls (n = 10)
**Emphysematous changes**	4 (66.7%)	7 (50%)	4 (40%)
**Vascular congestion**	4 (66.7%)	5 (35.7%)	3 (30%)
**Pneumonia**	2 (33.3%)	5 (35.7%)	6 (60%)
**Hemorrhage**	0 (0%)	3 (21.4%)	3 (30%)
**Pulmonary tumor**	2 (33.3%)	1 (7.1%)	0 (0%)
**Vascular abnormalities**	2 (33.3%)	0 (0%)	1 (10%)
**Fibrosis**	2 (33.3%)	1 (7.1%)	0 (0%)
**Normal lung tissue**	0 (0%)	1 (7.1%)	1 (10%)

## Data Availability

The original contributions presented in this study are included in the article. Further inquiries can be directed to the corresponding authors.
